# Knowledge, Acceptance, and Uptake of Family Planning: A Cluster Randomized Controlled Trial of Group Antenatal Care in Ghana

**DOI:** 10.3390/ijerph21081025

**Published:** 2024-08-03

**Authors:** Ruth Zielinski, Samia Abdelnabi, Georgina Amankwah, Vida A. Kukula, Veronica Apetorgbor, Elizabeth Awini, John Williams, Cheryl Moyer, Bidisha Ghosh, Jody R. Lori

**Affiliations:** 1 Department of Health Behavior and Biological Sciences, University of Michigan School of Nursing, 400 North Ingalls, Ann Arbor, MI 48109, USA; sabdelna@med.umich.edu (S.A.); bidisha@med.umich.edu (B.G.); jrlori@med.umich.edu (J.R.L.); 2 Dodowa Health Research Center, Ghana Health Service, Dodowa P.O. Box DD1, Greater Accra Region, Ghana; ginagyan@gmail.com (G.A.); vida.kukula@gmail.com (V.A.K.); veronica.apetorgbor@ghs.gov.gh (V.A.); awini.elizabeth@gmail.com (E.A.); williamsjeo@gmail.com (J.W.); 3 Department of Learning Health Sciences, University of Michigan Medical School, Ann Arbor, MI 48109, USA; camoyer@med.umich.edu

**Keywords:** antenatal care, family planning, Ghana, group antenatal care, group care, sub-Saharan Africa

## Abstract

The use of family planning (FP) methods significantly contributes to improved outcomes for mothers and their offspring. However, the use of FP remains low, particularly in low- and middle-income countries. A cluster randomized controlled clinical trial was implemented in Ghana, comparing group antenatal care (ANC) with routine care. The group ANC intervention included eight meetings where the seventh group meeting incorporated information and discussion regarding methods of FP. Data collection occurred at five time points: baseline (T0), 34 weeks’ gestation (T1), 6–12 weeks post birth (T2), 5–8 months post birth, and 11–14 months post birth (T4). At T1, there was a significantly greater increase in the knowledge of FP methods as well as the intention to use FP after the birth among the intervention group. The uptake of FP was significantly higher in the intervention group for all post-birth timepoints except for T4 where the control group had significantly higher rates. The reasons for the diminishing effect are unclear. An increasing uptake of FP methods requires a multifaceted approach that includes increasing accessibility, knowledge, and acceptability as well as addressing societal and cultural norms.

## 1. Introduction

In 2020, a striking statistic reported a maternal death almost every two minutes [1]. Each day, approximately 800 women globally lose their lives due to complications associated with pregnancy and childbirth that could often be prevented [1]. While over the past 20 years, there has been a 34% reduction in maternal deaths worldwide, the vast majority (95%) of maternal deaths are still occurring in low- and middle-income countries (LMICs) [1].

A woman from a low-income country is estimated to be 130 times more likely to die from a maternity-related cause compared to a woman from a high-income country [1]. As of 2020, sub-Saharan Africa (SSA) and Southern Asia combined were linked to approximately 87% of all maternal deaths worldwide, and of those deaths, nearly 70% took place in sub-Saharan Africa alone [1]. Thus, although there has been a reduction in maternal deaths globally, sub-Saharan Africa remains the region with the highest rates of maternal mortality.

The leading reasons for these maternal deaths stem from a range of complications associated with pregnancy and childbirth [2]. These encompass risks such as complications from unsafe abortions, infectious diseases, hemorrhagic events, hypertensive disorders, problematic labor and delivery, and indirect complications that arise due to pre-existing medical conditions influenced by pregnancy [1,2,3]. Factors contributing to this disparity include limited access to quality health care, a shortage of skilled health workers, widespread poverty, climate and humanitarian crises, and a lack of respectful, quality care hindering women’s willingness to seek necessary health care—the last factor being particularly prevalent in SSA [1,2,3].

Lowering the rates of unintended pregnancies and properly spacing births may help reduce maternal mortality and morbidity. Each year, 121 million women globally face unintended pregnancies, leading to 73 million induced abortions, of which 45% are unsafe [4]. These unsafe abortions result in 4.7–13.2% of maternal deaths annually [4]. Alarmingly, 97% of these unsafe abortions occur in LMIC countries, with nearly half taking place under precarious conditions in Africa [4].

Worldwide fertility rates have gradually declined in recent decades to 2.5 total births per woman [5]. African countries, specifically those in SSA, have experienced a slower decline and are projected to produce more than half of livebirths globally by 2100 [5]. In Ghana, fertility rates have decreased by 1.9 births per woman to a current average of 3.5 births per woman [6].

While contraceptive use has increased globally, meeting 77.5% of family planning (FP) needs, progress in SSA has been comparatively slower [7]. Despite a recent increase in contraceptive use in SSA, the current usage rate is still relatively low, with only 33% of women having used contraception in 2020 [7]. In Ghana, only 30% of FP needs are fulfilled [7]. Therefore, despite progress in FP use, there remains an urgent need to address the issues around contraception and FP, particularly in regions like SSA, for the better health and well-being of women and their offspring.

Antenatal care (ANC) visits are an optimal time to discuss FP, but many women do not attend antenatal visits regularly and visits are often limited to risk assessment. Group care is an innovative approach to antenatal care whereby pregnant women with similar due dates receive care in group meetings. Each meeting includes a topic that aligns with the gestation of the pregnancy. While most studies have been conducted in the United States, there is evidence that group ANC is both feasible and beneficial within the context of LMICs [8]. Most studies have indicated increased FP knowledge and uptake among women participating in group ANC [9]. There is preliminary evidence from an observational study in Ghana that the group model of ANC increases the uptake of FP [10]. Each meeting has an educational topic for discussion. For this study, the group ANC curriculum included FP methods and use in the seventh meeting when women were in the third trimester of pregnancy. The purpose of this study, using a cluster randomized control design, was to assess if this model of group ANC that included information and a discussion of FP methods would increase the acceptance, knowledge, and uptake of FP.

## 2. Materials and Methods

### 2.1. Theoretical Framework

This study used a theoretical model originally developed by Squiers et al. and modified in our preliminary research to assess maternal health literacy [11]. The Health Literacy Skills Framework uses an ecological perspective to develop and test potential interventions to impact a patient’s health literacy. Our modified framework addresses how group ANC builds knowledge by increasing the comprehension of stimuli, promoting self-determination, increasing action, and ultimately improving maternal health behaviors and outcomes. It considers how the individual’s comprehension of stimuli and potential mediators may impact health behaviors and outcomes. The modified theoretical model was renamed Maternal Health Literacy Skills Framework [12] and guided the aims and data analytic plan.

### 2.2. Design

A five-year cluster randomized controlled trial was conducted among 14 health facilities in Ghana, with detailed methodology published elsewhere [13]. Cluster randomized controlled trials (cRCTs) are well suited to testing differences in a method or approach to patient care. This approach is better able to evaluate whether a new standard of care, guideline recommendation, or other practice-wide, hospital-wide, or system-wide change affects patient outcomes. When there is a significant potential for contamination in the study if a traditional randomized controlled trial is used, a cRCT may be a preferred approach [14].

### 2.3. Sampling

Facilities were paired according to similar characteristics, such as facility type, district, and number of monthly ANC registrants, to ensure that each pair was similar in these criteria. Each matched pair of facilities was randomly assigned to group ANC (intervention) or to routine, individual ANC (control).

### 2.4. Setting

The study encompassed four districts within the Eastern Region of Ghana. This region, positioned north of the Greater Accra Region, has a fertility rate similar to the national average in sub-Saharan Africa [4].

### 2.5. Participants

Women presenting to care at the 14 participating health facilities were recruited with the assistance of clinic staff and trained Research Assistants (RAs). Inclusion criteria included pregnancy with a gestation of less than 20 weeks, language proficiency in Dangme, Ga, Akan, Ewe, or English, being above 15 years of age, and having a low risk pregnancy. Inclusion criteria were selected to be as inclusive as possible and enroll women early in pregnancy to be able to complete the full dose of the intervention. The study duration of 36 months allowed for at least four cohorts of participants to move through ANC and into the postnatal period. Trained RAs approached potential participants with information about the study, obtained informed consent, and conducted baseline data collection.

### 2.6. Intervention

**Figure 1:**
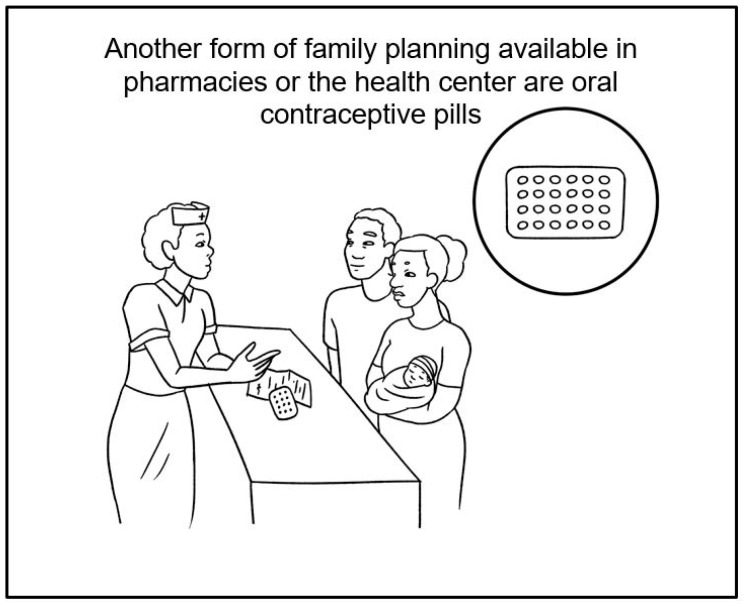
Example of a picture card used in group antenatal care Meeting 7.

Each group ANC meeting introduced a new topic, with the topic of Meeting 7 being FP. The group ANC model of care was designed to be interactive rather than didactic. The FP discussion began with a story designed to initiate discussion around family spacing and FP. Methods of FP were introduced and discussed using picture cards (Figure 1). The group were asked to indicate whether they believed certain statements, such as “Vasectomy will decrease a man’s sexual ability”, to be a myth or truth. Lastly, there was a demonstration of the proper use of condoms.

### 2.7. Data Collection

**Figure 2:**
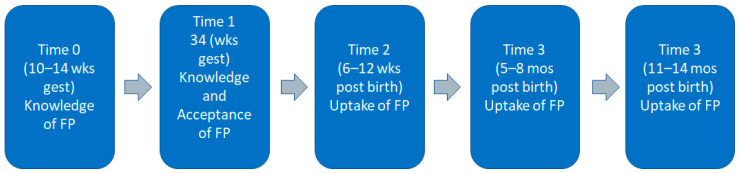
Timeline of family planning data collection.

Data collection for FP variables occurred at five time points (Figure 2): baseline (T0), 34 weeks’ gestation (T1), 6–12 weeks post birth (T2), 5–8 months post birth, and 11–14 months post birth (T4). Data were collected directly from participants rather than through chart review as there were limited patient data in facility medical records in Ghana. At the time of data collection, medical records were paper copies only, and most of the pertinent data were kept by the woman in a Maternal Health Record booklet that she then brought to the facility. Data were collected using REDCap, a secure web application, and surveys were administered by RAs using password-protected tablets capable of offline data storage. Demographic information was collected at baseline. Data on the uptake of FP were collected at all timepoints post birth.

Knowledge was assessed by asking participants whether they believed pregnancy spacing to be important (yes/no) and to recall the FP methods they knew of. The number of FP methods participants could recount was then recorded numerically by the RAs. For example, if they were able to identify condoms and pills, the number “2” was recorded by the RAs. FP data collection for the evaluation of knowledge was performed at baseline and 34 weeks’ gestation, the time point for data collection that occurred after Meeting 7, where FP was introduced and discussed. Acceptance was assessed by asking participants about their intention to use FP (yes/no). Intention to use FP was assessed at baseline (T0), 34 weeks’ gestation (T1), 6–12 weeks post birth (T2), and 11–14 months post birth (T4). The uptake of FP was assessed at baseline (T0), 6–12 weeks post birth (T2), 5–8 months post birth (T3), and 11–14 months post birth (T4) by participants self-reporting any FP methods.

### 2.8. Analysis

Data management was carried out using SAS Version 9.4 (Manufacturer: SAS Institute, 100 SAS Campus Drive, Cary NC 27513-2414, USA) and data analysis was conducted using Stata Version 17.0 (Manufacturer: StataCorp LLC, 4905 Lakeway Drive, Colege Station, TX 77845-4512, USA). Results from T0 and T1 were compared between group ANC (intervention) and routine ANC (control) to assess the efficacy of group ANC in improving the FP knowledge scores over time. The FP knowledge scale is an 11-point composite scale based on their knowledge of the different FP methods. A Poisson regression adjusted for clustering was run to compare the two groups across the two time points. Additional analyses were performed to evaluate the groups’ intention to use FP and the uptake of any FP method over time. The intention to use FP, as well as the uptake of any FP method, were both yes/no questions. Therefore, a logistic regression adjusted for clustering was conducted to evaluate both questions.

## 3. Results

### 3.1. Demographics

Table 1 presents the sample demographic characteristics, demonstrating the comparability of the intervention and control groups across key variables. No significant differences were observed between the intervention or control group in demographic characteristics (maternal age, education, religion, first pregnancy, location of delivery, wealth index, number of prior pregnancies, or place of delivery). Most of the participants (96%) were married or cohabitating. The majority of participants indicated a middle or higher level of education, identified themselves as Christians, and were not experiencing their first pregnancy, indicating a relatively homogeneous study population.

### 3.2. Family Planning Knowledge

**Figure 3:**
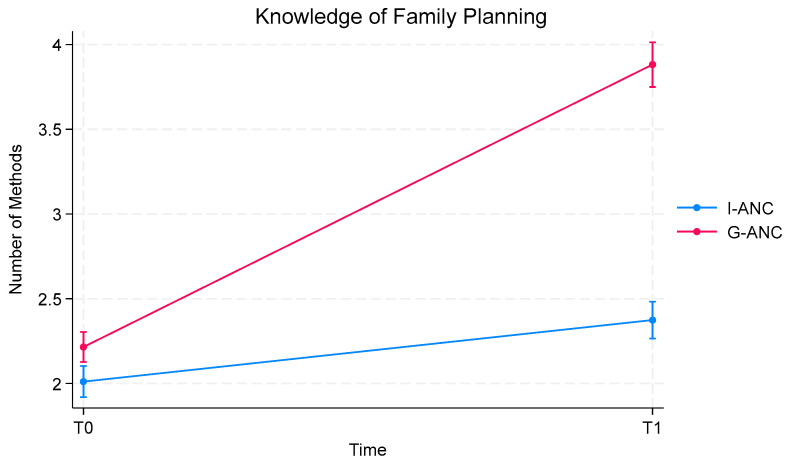
Participant knowledge of family planning methods from Time 0 to Time 1 by group ANC and individual ANC.

Women understood the importance of pregnancy spacing, with no difference between the groups (>97% in both groups). Participants in both groups could recall a similar number of FP methods at baseline data collection (mean = 2.2 for group ANC vs. 2.0 for control). At T1, however, the group ANC participants had a significantly higher increase in FP knowledge (Figure 3), with a mean score of 3.9 FP methods compared to a mean score of 2.4 for the control group (*p* < 0.0001).

### 3.3. Family Planning Acceptance

**Figure 4:**
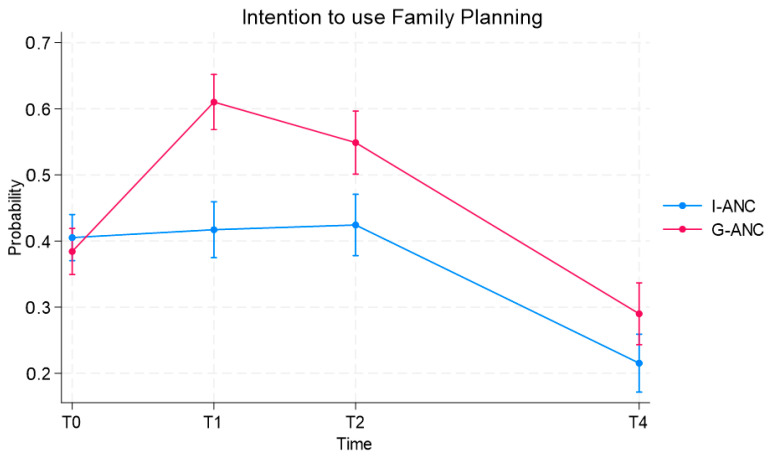
Intention to use family planning by group ANC and individual ANC.

To assess the acceptance of FP, participants were asked at T0 (10–16 weeks’ gestation), T1 (approximately 34 weeks’ gestation), T2 (6–12 weeks post birth) and T4 (11–14 months post birth) if they intended to use FP post birth (yes/no). The percentage of women in group ANC intending to use FP increased from 40.5% at baseline to 61.0% at T1, dropping to 55% at T2 and 29% at T4. The percentage of women in the control group who intended to use FP post birth remained essentially unchanged at approximately 40% from T0 to T2, dropping to 29% at T4 (Figure 4). This change in the intention to use FP is significantly different for the two groups over time (*p* < 0.0001).

### 3.4. Family Planning Uptake

**Figure 5:**
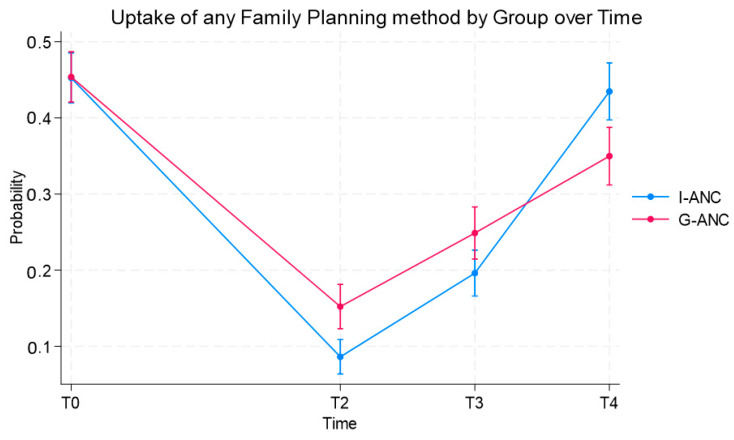
Uptake of any family planning method by group ANC and individual ANC.

The uptake of any method of FP was assessed at T0 (10–16 weeks’ gestation), T2 (6–12 weeks post birth), T3 (5–8 months post birth), and T4 (11–14 months post birth) as shown in Figure 5. The FP uptake significantly differed for the two groups over time (*p* < 0.0001). For both groups, the uptake of FP was low at T2 but significantly higher for the intervention group (8.6% vs. 15.0). At T3 (5–8 months post birth), significantly more women indicated that they were using FP in the intervention group (25% vs. 20%). However, at T4 (11–14 months post birth), while both groups had an increase in FP uptake compared to T2, significantly more women in the control group were using FP (43% vs. 35% for the intervention group). At T4, the most commonly used contraception method in both groups was injectable (Medroxyprogesterone acetate), followed by contraceptive implants (Table 2).

## 4. Discussion

The existing literature suggests that a multitude of factors contribute to whether individuals use FP. These include misinformation, societal influences, and acceptability reasons [15,16]. Additionally, the pace at which FP methods are adopted could be influenced by the promotion and accessibility of FP services, the community’s acceptance and cultural views on modern contraception methods, and the general understanding of these methods [17,18,19,20].

In SSA, many women indicate that a lack of knowledge or incorrect information concerning FP is a significant barrier [21,22]. This lack of knowledge often stems from a multitude of sources, such as inadequate health education, gaps in communication, and sometimes cultural or societal norms that may discourage open discussions about sexual health and FP [21,22,23]. Mistrust in the safety and effectiveness of contraceptives compounds these challenges. Myths and misconceptions surrounding certain contraceptive methods can lead to anxiety and apprehension towards their use. For instance, fears about potential side effects, future fertility, or beliefs that contraceptives can cause health problems are not uncommon [16,20,23]. Unfortunately, such misinformation often deters women from using FP services even when available. In this study, we found that participants knew the importance of family spacing in improving the health of mothers and infants. While knowledge of FP methods among participants was low at baseline, women in group ANC demonstrated a greater increase in knowledge. In the small group setting, the seventh meeting included not only information about FP methods but also provided discussion time to help dispel myths and fears surrounding FP.

Knowledge, while important, is not sufficient to increase the uptake of FP. Societal influences, too, play a significant role. In many societies, including those in sub-Saharan Africa, traditional beliefs and attitudes towards FP and reproductive health can greatly impact a woman’s choice and use of contraceptives. Pressure from family, partners, or the community to bear children or to abstain from using contraceptives can often supersede the woman’s preferences or needs [16,23,24,25,26]. Moreover, societal stigma associated with contraceptive use could also make women less likely to seek or utilize FP services [24,26,27,28].

In Ghana, the rate of modern contraception usage stands at 27.8%, indicating a considerable gap in FP method utilization [29]. Despite a global rise in contraception use, many individuals still do not employ these methods to prevent unintended pregnancies. Rates of FP in this study were similar to the national rates in Ghana, with less than half of the participants indicating that they were using FP at T4 (11–14 months post birth). While participants in group ANC had an increase in uptake at T3, significantly more women in the control group were using FP at T4. The reason for this is unclear and warrants further investigation. Continuing group care into the post-natal period has been implemented successfully in Malawi with promising results [30]. Rather than one meeting during pregnancy, FP could be revisited when the topic is more salient.

## 5. Limitations

Participants were assessed based on their ability to recall different methods, rather than on the depth of their knowledge about those methods. The uptake of FP was measured by participant reports rather than chart reviews. Reasons for not using FP were not collected and would be an important part of future studies.

## 6. Conclusions

Group ANC shows promise in improving maternal and newborn outcomes in LMIC [4]. In this study, there was a significant increase in knowledge and acceptability regarding FP, both important components in improving the use of FP. While there was an uptake in FP among those participating in group ANC, the effects did not extend to 12 months, indicating that the effect may not be sustained in the long term. Improving the adoption and use of FP methods in regions like sub-Saharan Africa requires a multifaceted strategy [30]. Strategies encompass not only ensuring the availability and accessibility of FP services but also providing accurate and comprehensive information about contraceptive methods, addressing cultural misconceptions, and promoting an open and supportive societal attitude towards FP. Extending group care beyond the antenatal period into the postnatal year may be beneficial in improving outcomes, including the use of FP [31]. Further studies of group care that extends post birth are needed.

## Figures and Tables

**Table 1:** Participant demographic information.

Categorical Variables n (%)	Overall	Control (I-ANC)	Intervention (G-ANC)	*p*-Value
	N = 1761 *	N = 884	N = 877	
Age Category				
Less than 25	501 (28%)	266 (53%)	235 (47%)	0.193
25–34	987 (56%)	477 (48%)	510 (52%)	
35 or more	273 (16%)	141 (52%)	132 (48%)	
Maternal Education				
Primary	246 (14%)	120 (49%)	126 (51%)	0.6895
Middle/JHS/JSS	829 (49%)	429 (52%)	400 (48%)	
Secondary/SHS/Technical/Vocational	459 (27%)	223 (49%)	236 (51%)	
Tertiary	164 (10%)	83 (51%)	81 (49%)	
Partner Education				
Middle/JHS/JSS or less	666 (39%)	335 (50%)	331 (50%)	0.8525
Secondary	627 (37%)	306 (49%)	321 (51%)	
Tertiary	261 (16%)	126 (48%)	135 (52%)	
N/A, Unknown	137 (8%)	64 (47%)	73 (53%)	
Religion				
Christian	1646 (93%)	835 (50.4%)	811 (49.6%)	0.1185
Muslim	97 (6%)	39 (39%)	58 (61%)	
Other	18 (1%)	10 (56%)	8 (44%)	
First Pregnancy				
No	1412 (80%)	703 (50%)	709 (50%)	0.4876
Yes	349 (20%)	181 (52%)	168 (48%)	
Location of Delivery				
Hospital/Polyclinic/Health Center	1711 (97%)	853 (50%)	858 (50%)	0.0904
Other	50 (3%)	31 (62%)	19 (38%)	
Continuous Items: Mean (SD)				
Maternal age	28.2 (5.8)	28.1 (6)	28.3 (5.6)	0.5042
Wealth index	6.8 (2.4)	6.9 (2.4)	6.9 (2.3)	0.6174
Number of previous pregnancies	3.5 (1.4)	3.5 (1.4)	3.5 (1.5)	0.7075

* This is the total sample size. If it does not add across the demographics, then it is missing. Categorical variables were compared using a chi-square test. Maternal age and wealth index were tested using a two-sample *t*-test. The number of previous pregnancies was tested using a Mann–Whitney Wilcoxon test (non-parametric).

**Table 2:** Family planning methods used at Time 4.

Category	Method	I-ANC (N = 688)	G-ANC (N = 626)	Total (N = 1314)
	None	393 (57%)	412 (65.7%)	803 (61.1%)
Non-pharmacologic	Lactation amenorrhea (LAM)	15 (2.2%)	1 (0.2%)	16 (1.2%)
	Cycle beads	39 (5.8%)	20 (3.2%)	59 (4.5%)
	Withdrawal	1 (0.2%)	2 (0.3%)	3 (0.2%)
	Condoms	6 (0.9%)	8 (1.3%)	14 (1.1%)
Pharmacologic	Pills/oral contraception	41 (6%)	23 (3.7%)	64 (4.9%)
	Injectable	117 (17%)	79 (12.6%)	196 (15%)
	Emergency contraception	6 (0.9%)	1 (0.2%)	7 (0.5%)
Long-acting reversible	Implants	48 (7%)	45 (7.2%)	93 (7.1%)
	Intrauterine devices	1 (0.2%)	13 (2.1%)	14 (1.1%)
Permanent	Sterilization (female)	18 (2.6%)	8 (1.3%)	26 (2%)
	Sterilization (male)	0 (0)	0 (00	0 (0)
Other	Other	3 (0.4%)	14 (2.2%)	17 (1.3%)

## Data Availability

Data are available from University of Michigan Deep Blue Data: https://deepblue.lib.umich.edu/data/catalog?f%5Bcreator_sim%5D%5B%5D=Moyer%2C+Cheryl&locale=en (accessed on 28 July 2024).
